# Comparative safety and efficacy of percutaneous radiofrequency thermocoagulation and percutaneous balloon compression in CT-guided and local anesthesia for recurrent trigeminal neuralgia

**DOI:** 10.3389/fneur.2023.1336261

**Published:** 2024-01-05

**Authors:** Lulu Xi, Xiaohui Liu, Hongchen Shi, Wenbiao Han, Liqin Gao, Li Wang, Junpeng Liu, Yue Ren, Yuanyuan Du, Guangzhao Liu

**Affiliations:** ^1^Department of Pain, Second Hospital of Hebei Medical University, Shijiazhuang, Hebei, China; ^2^Department of Neurology, Second Hospital of Hebei Medical University, Shijiazhuang, Hebei, China

**Keywords:** percutaneous balloon compression, percutaneous radiofrequency thermocoagulation, CT-guided, recurrent, trigeminal neuralgia, local anesthesia

## Abstract

**Background:**

There are several ways to treat trigeminal neuralgia (TN); however, TN may recur after treatment. Although microvascular decompression (MVD) is considered an effective treatment for trigeminal neuralgia, patients with recurrence may not be willing to undergo craniotomy.

**Objective:**

This study compared the safety and efficacy of percutaneous radiofrequency thermocoagulation and percutaneous balloon compression for treating recurrent trigeminal neuralgia.

**Methods:**

This was a prospective non-randomized controlled study. A total of 52 with recurrent TN were scheduled to undergo surgery in our Hospital from January–June 2021. The patients were classified into percutaneous radiofrequency thermocoagulation (PRT) and percutaneous balloon compression (PBC) groups based on the treatment. All surgeries were performed under computed tomography guidance and local anesthesia. Post-operative complications were also observed. Pain was assessed using the visual analog scale (VAS) and Barrow Neurological Institute (BNI) scale. Efficacy indices were evaluated at 3, 6, 12, and 18 months after surgery.

**Results:**

During follow-up, the efficacy rates of the two methods within 18 months were 76.0 and 88.9%, respectively. All patients had hypoesthesia on the affected side, and no severe complications. Notably, 5 patients (20%) in the PRT group with multiple-branch pain, including the first branch of the trigeminal nerve (V1) pain in the PRT group, received radiofrequency therapy for the supraorbital notch (foramen) after puncture of the foramen ovale. However, multiple pain episodes resolved with only one operation in the PBC group.

**Conclusion:**

CT-guided percutaneous radiofrequency thermocoagulation and percutaneous balloon compression under local anesthesia may be good options for treating recurrent trigeminal neuralgia. Percutaneous balloon compression may be recommended when multiple branches are involved, particularly in cases of V1 neuralgia.

## Introduction

Trigeminal neuralgia (TN) is one of the most painful diseases, with an annual incidence of 12.6/100,000–27/100,000 (1, 2). It is characterized by recurrent, unilateral, temporary, and severe electrocute-like pain with a rapid onset and short duration (up to several minutes) (3). According to the international classification of headache disorders 3^rd^ edition (ICHD-3), TN can be classified into three subgroups: classic, secondary, and idiopathic TN (4). Several treatments for classical TN, such as percutaneous radiofrequency thermocoagulation (PRT), percutaneous balloon compression (PBC), and microvascular decompression (MVD), have been proven effective; however, previous studies have shown that even patients who undergo the most durable option, MVD, will typically eventually relapse if followed up long enough (5). MVD is currently considered to have the lowest recurrence rate, and one study demonstrated that MVD resulted in a greater percentage of pain-free status at 36 months than PRT (6). Another study with 2–3 years of follow-up showed that significantly more patients were completely pain-free after MVD compared with PBC (7). However, when pain recurs, patients are often reluctant to undergo another craniotomy, and MVD is unsuitable for elderly patients or those with poor health. Therefore, PRT or PBC may have been selected.

PBC and PRT are the most common treatments for TN in the Pain Department; however, a high recurrence rate is also a problem. The recurrence rate of PRT is 46% within 5 years, and that of PBC is 19.2% (8, 9). A comparison between the two types of surgery has been reported for primary TN but not for patients with relapse (10). Therefore, this study compared the efficacy and safety of PRT and PBC in 52 patients with recurrent TN who previously received different treatments.

## Methods

### Study design and participants population

This was a prospective non-randomized controlled study. From January 2021–June 2021, we enrolled 52 patients with recurrent TN at the Pain Department of our Hospital. The patients were diagnosed with classic trigeminal neuralgia and pain involving one or more branches. Written consent was obtained from all participants in this study. All surgeries were performed by the same surgeon, and the medical records of all patients were collected and retrospectively analyzed. The study was conducted following the Declaration of Helsinki and approved by the Ethics Committee of our hospital (2020-R562).

**Figure 1:**
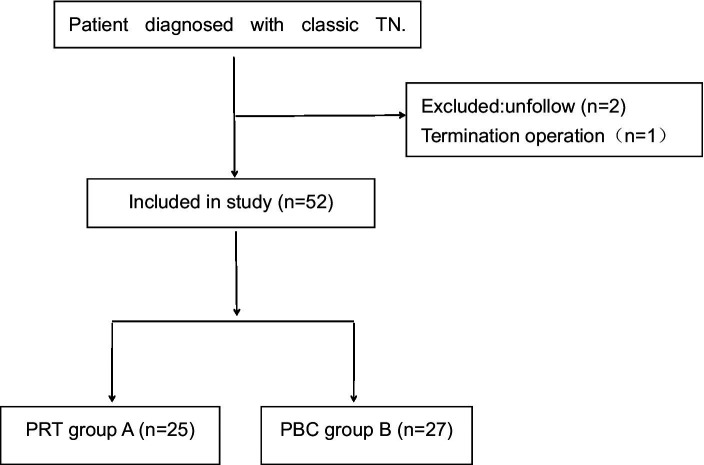
Flow chart of inclusion of trigeminal neuralgia patients.

A total of 52 patients were classified into the PRT (25 patients) and PBC (27 patients) groups. A flowchart of the entry and exit of patients is shown in Figure 1. All surgeries were performed under computed tomography guidance and local anesthesia. We developed the inclusion and exclusion criteria for patients preparing for surgery, as shown in Table 1.

**Table 1:** Inclusion criteria and exclusion criteria.

Inclusion criteria	Exclusion criteria
Definite diagnosis of classic TN Age between 30 and 90 VAS ≥ 4 points TN patients who had a history of TN related surgery, such as MVD, PRT, GKRS and PBC	Age below 30 years old or above 90 years old Local infection of the puncture site Coagulopathy or hemorrhagic disease mental illness cannot cooperate with the operation Severe heart, brain, lung and liver disease

TN, Trigeminal Neuralgia; VAS, Visual analog score; MVD, microvascular decompression; PRT, percutaneous radiofrequency thermocoagulation; GKRS, gamma knife radiosurgery; PBC, Percutaneous Balloon Compression.

### Surgery

This study performed PRT and PBC under computed tomography (CT) guidance and local anesthesia. Patients were not required to fast before surgery; however, intravenous fluids were administered to ensure intravenous drugs were administered when necessary. CT helps in improving puncture accuracy. Therefore, we scanned the position of the puncture needle using CT and reconstructed the anatomical image of the skull base of the patient by three-dimensional CT imaging technology. The relationship between the puncture tip and the anatomy of the skull base was visible.

#### PBC

**Figure 2:**
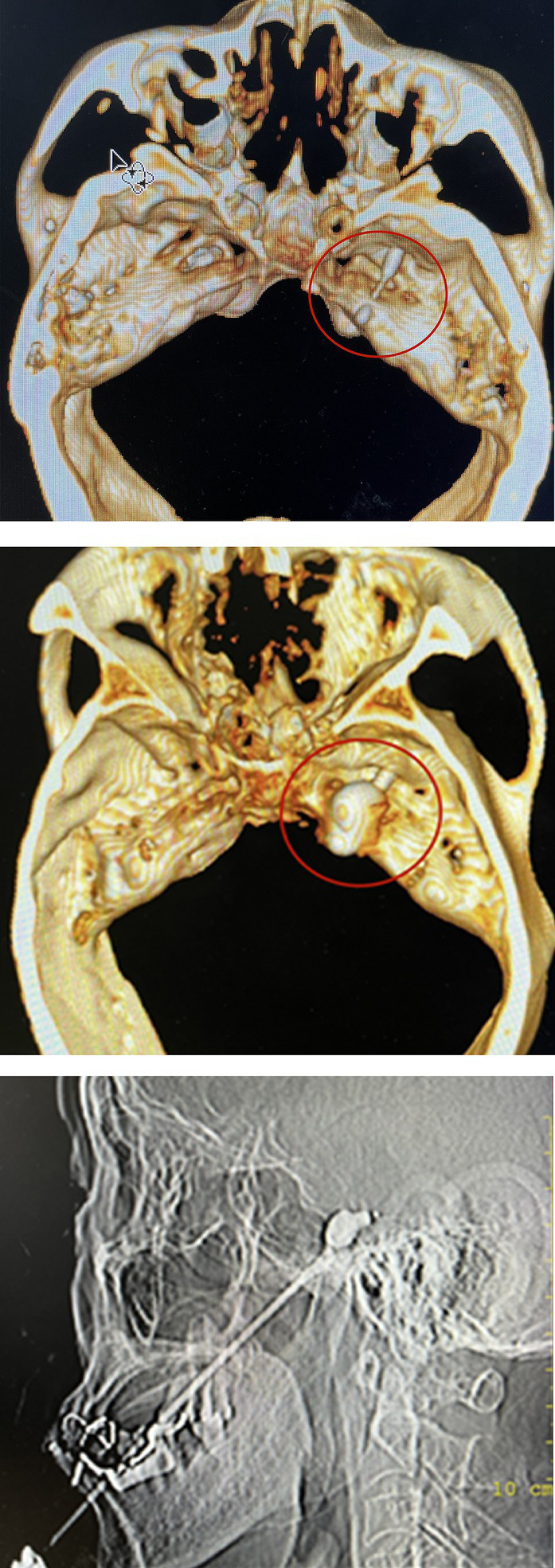
The location of balloon catheter can be determined by inserting balloon catheter under CT-guided. After the iohexol was injected, a “pear shaped image” was displayed on lateral CT imaging, indicating successful surgery.

The patient remained awake during the operation and was placed in the supine position with the head tilted backward. ECG monitoring was routinely performed to monitor blood pressure, heart rate, and oxygen saturation. Furthermore, midazolam (0.03 mg/kg) was injected intravenously for sedation before surgery. A 22G local anesthesia needle, 10 cm in length, was used for a puncture, and 1% lidocaine (0.5 mL) was injected into the trigeminal nerve ganglia of the foramen ovale under CT guidance. Then a 14G balloon puncture needle was inserted along the original path. The needle tip entered the foramen ovale, and a No. 4 Fogarty balloon catheter was inserted. After confirming the correct location, 0.5–0.8 mL iodohexyl was injected into the catheter to inflate the balloon. The balloon and its position were adjusted during the lateral CT scan of the skull until a pear-shaped image was obtained, as shown in Figure 2. The catheter was removed after applying pressure for 3 min.

#### PRT

**Figure 3:**
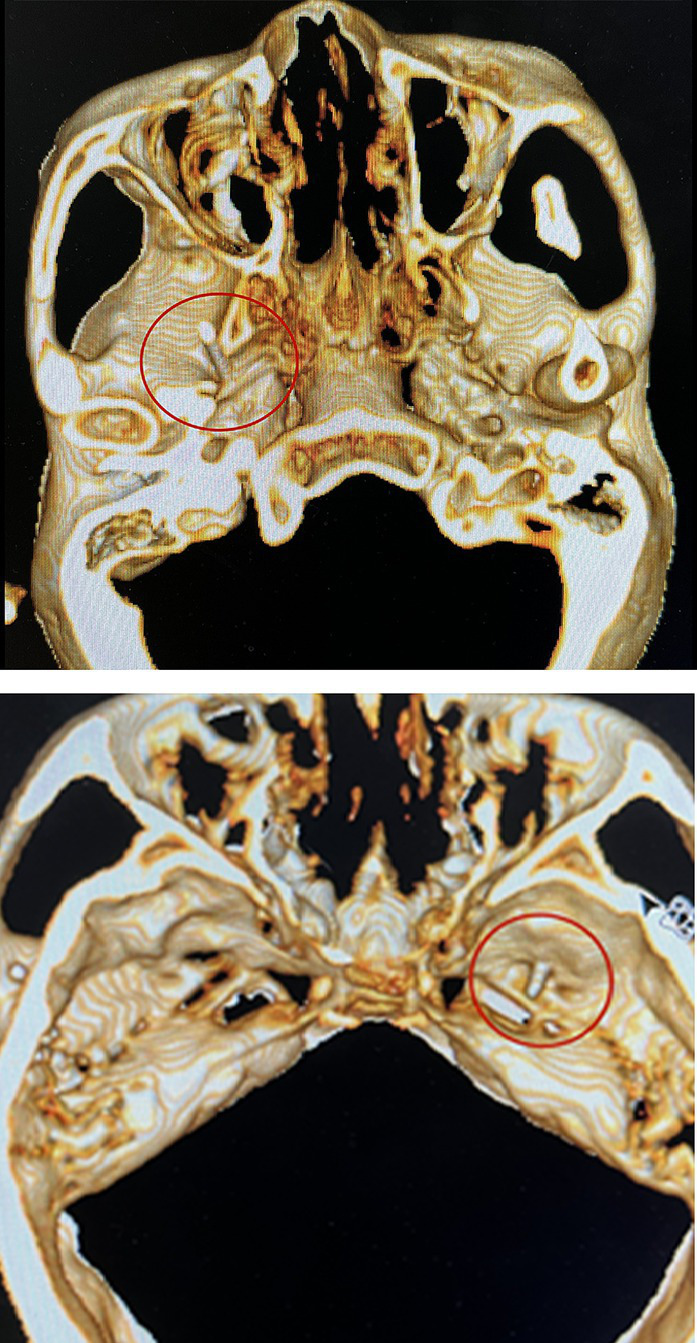
Radiofrequency trocar was used to enter the foramen ovale under the guidance of CT, and the radiofrequency machine was connected. Sensory stimulation at 50HZ and motor stimulation at 2HZ were given. After confirming the location, the patient was given three 90-s radiofrequency thermocoagulation at 80°C.

The patient was placed in the supine position at the back of the head. Blood pressure, heart rate, and oxygen saturation were routinely measured. Before surgery, 0.03 mg/kg midazolam was injected intravenously for sedation. A 10 cm, 22G radiofrequency trocar was used to enter the foramen ovale under CT guidance, and the radiofrequency machine was connected. Sensory stimulation at 50 Hz and motor stimulation at 2 Hz were applied. An abnormal sensation or muscle contraction in the non-diseased area required adjustment of the tip position until the stimulated area covered the painful area, as shown in Figure 3. In addition, local anesthesia of 0.2 mL, 2% lidocaine injection was given. When the pain area became numb, the patient was given three 90-s radiofrequency thermocoagulation at 80°C.

Notably, for the five patients (20%) in the PRT group who had multiple-branch pain, including the first branch of the trigeminal nerve (V1) pain, we performed radiofrequency therapy of the supraorbital notch (foramen) after the puncture of the foramen ovale. We observed that, even if the location of the foramen ovale puncture was accurate, it was difficult to induce an abnormal sensation in the V1 nerve region, which may be related to the anatomy of the V1 nerve. Nevertheless, supplementary radiofrequency therapy of the supraorbital nerve is required to ensure a therapeutic effect.

**Figure 4:**
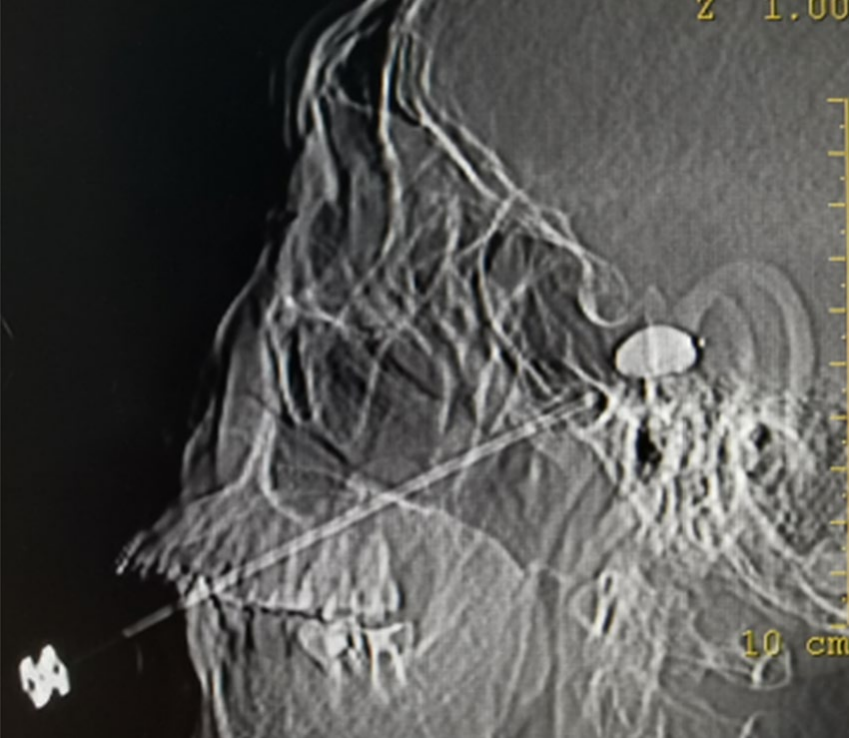
The pear image does not appear. It indicates no pressure on trigeminal ganglion.

In the PBC group, one patient still experienced postoperative pain, which may be related to the absence of a standard pear shape during the operation, as shown in Figure 4. PBC was administered again 10 days later, and the pain resolved.

### Therapeutic assessment and follow-ups

The Barrow Neurological Institute (BNI) and visual analog scale (VAS) were used to assess the degree of pain. Complications and pain recurrences were also recorded. The Barrow Neurological Institute (BNI) assessed pain intensity in patients, as shown in Table 2. The BNI pain intensity scale (BNI Pain Score I–II) and a VAS score < 4 were considered effective. Patients were followed-up for 3, 6, 12, and 18 months after surgery, and their pain and numbness were recorded.

**Table 2:** Barrow Neurological Institute (BNI) Pain Intensity Scale.

Score and description	
I	No pain, no medication
II	Occasional pain, not requiring medication
III	Some pain, adequately controlled with medication
IV	Some pain, not adequately controlled with medication
V	Severe pain, no pain relief

### Statistical analysis

SPSS 24 software was used for statistical analyses. All variables were normally distributed, and quantitative variables are presented as mean ± standard deviation. An independent *t*-test was used to compare groups, and qualitative data were expressed as frequency and percentage (%); statistical significance was set at *p* < 0.05.

## Results

### General demographics

Out of the 52 patients, 23 were male, and 29 were female, ranging in age from 31 to 89 (mean 62.75 ± 16.41 years). The total disease course ranged from 6 to 480 months. Furthermore, 35 patients experienced pain on the right side and 17 on the left side. Moreover, 31 patients underwent PRT, 15 underwent PBC, 5 underwent MVD, and 1 underwent gamma knife radiosurgery (GKRS). Of the 52 patients, 3 had V1, 7 had V2, 15 had V3, 10 had V1 + V2, 16 had V2 + V3, and 1 had V1 + V2 + V3 pain. General demographic data are shown in Table 3.

**Table 3:** Patient demographics and clinical data.

Characteristics	Baseline (Mean ± SD)
Gender(n) Male Female	23 29
Age year Mean ± SD Range	62.75 ± 12.41 31–89
Pain duration, month Range	6–480
Pain side(n) Right Left	35 17
Type of prior procedure(n) MVD PBC GKRS PRT	5 15 1 31
Branches affected(n) V1 V2 V3 V1 + v2 V2 + V3 V1 + V2 + V3 Preoperative VAS	3 7 15 10 16 1 group A:7.88 ± 1.30 group B:7.66 ± 1.44

VAS, Visual analog score; MVD, microvascular decompression; PRT, percutaneous radiofrequency thermocoagulation; GKRS, gamma knife radiosurgery; PBC, Percutaneous Balloon Compression.

### Clinical outcome

There were no significant differences in age, sex, preoperative VAS score, and preoperative BNI score between the 25 patients in the PRT group and the 27 patients in the PBC group (*p* > 0.05). Furthermore, the blood pressure, heart rate, and oxygen saturation of all patients were stable during the operation, and there were no significant differences before and after the operation (*p* > 0.05). Five patients (20%) in the PRT group with multiple-branch pain, including V1 pain in the PRT group, received radiofrequency therapy for the supraorbital notch (foramen) after puncture of the foramen ovale. However, multiple pain episodes resolved with only one operation in the PBC group.

**Figure 5:**
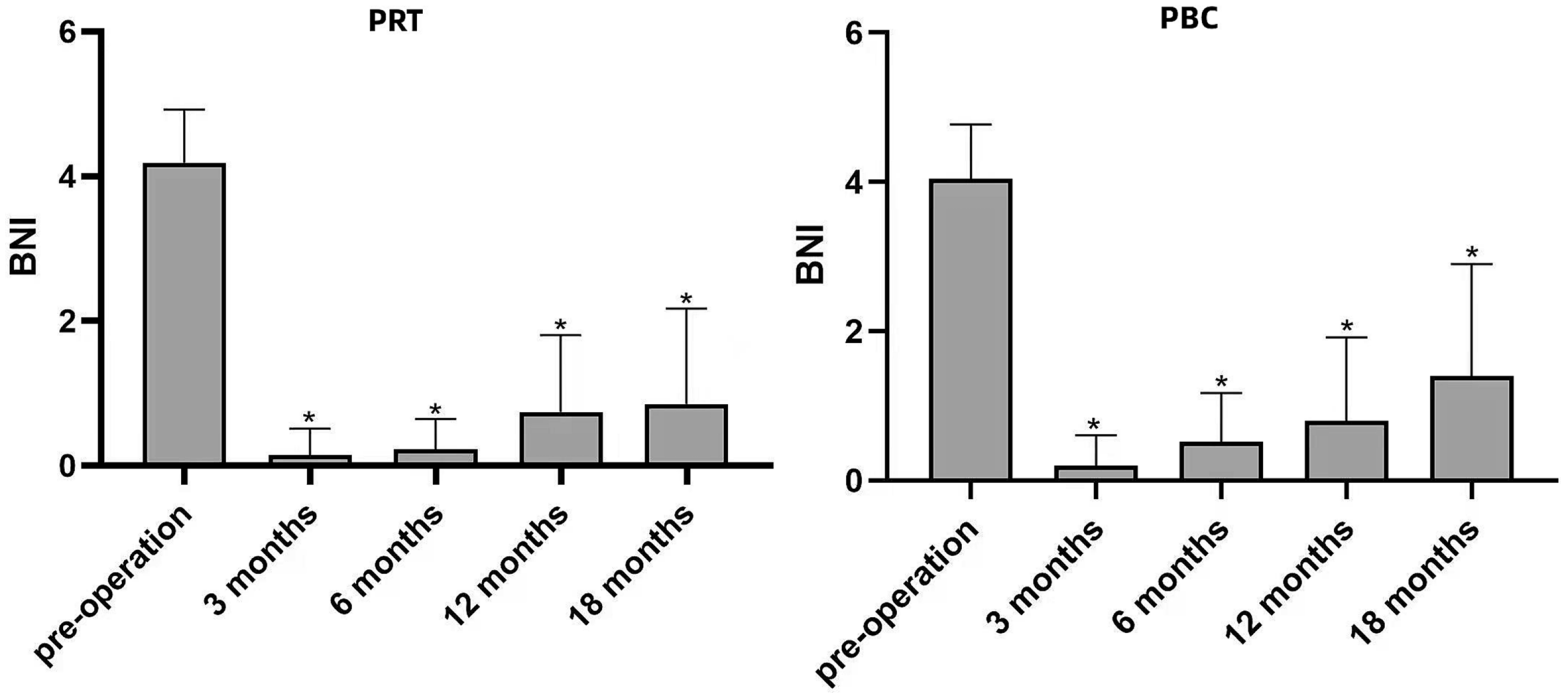
Changes in BNI scores after PRT and PBC compared with preoperative scores, *p < 0.05. BNI, Barrow Neurological Institute; PRT, Percutaneous Radiofrequency Thermocoagulation; PBC, Percutaneous Balloon Compression.

According to the BNI, the rate of pain relief (BNI I–II) within 6 months in both groups was 100%. In the 12 months of follow-up, there were 3 patients (12.0%) in the PRT group and 2 patients (7.4%) in the PBC group. Furthermore, 18 months after the operation, 6 patients were in the PRT group (24%), and 3 were in the PBC group (11.1%). There were significant differences in the BNI scores between the two groups before and after surgery (*p* < 0.05), as shown in Figure 5. However, there was no significant difference in the BNI scores between the two groups after surgery (*p* > 0.05).

After 18 months of follow-up, the effective pain control rates were 76.0 and 88.9% in the PRT and PBC groups, respectively. The mean VAS score of the PRT group was 7.88 ± 1.30 before surgery, 0.32 ± 0.62 at 3 months, 0.60 ± 0.91 at 6 months, 0.80 ± 1.38 at 12 months, and 1.28 ± 1.74 at 18 months. The mean VAS score of the PBC group was 7.66 ± 1.44 before surgery, 0.18 ± 0.48 at 3 months, 0.33 ± 0.55 at 6 months, 0.77 ± 1.05 at 12 months, and 0.70 ± 1.32 at 18 months. There was no significant difference in VAS scores between the PRT and PBC groups during the follow-up period. The VAS scores are presented in Table 4.

**Table 4:** Comparison of VAS score between the 2 groups.

	Group A	Group B	*p*-value
3 months	0.32 ± 0.62	0.18 ± 0.48	0.923
6 months	0.60 ± 0.91	0.33 ± 0.55	0.717
12 months	0.80 ± 1.38	0.77 ± 1.05	0.998
18 months	1.28 ± 1.74	0.70 ± 1.32	0.677

Notably, a patient experienced decreased blood pressure, heart rate, and sweating during PBC. The patient complained of palpitations, and supportive treatment was provided. Consequently, the symptoms disappeared within approximately 30 min. Therefore, the operation was terminated to ensure safety.

### Side effects and complications

Postoperative hypoesthesia of the face and mucosa was observed in all the patients. After surgery, 3 patients (12.0%) in the PRT group and 12 patients (44.4%) in the PBC group developed masticatory fatigue. Nevertheless, the degree of masticatory fatigue was mild, and all patients recovered within 3 months. Within 24 h after surgery, a patient (4.0%) in the PRT group had a headache, 2 patients (8.0%) had nausea or vomiting, 9 patients (33.3%) in the PBC group had a headache, and six patients (22.2%) experienced nausea and vomiting. Although none of the patients in the PRT group developed dizziness, 5 patients (18.5%) in the PBC group developed dizziness. Nevertheless, all symptoms disappeared the day after surgery. Notably, 5 patients (18.5%) with diplopia were in the PBC group; however, they returned to normal after 6 h. This may be related to the use of local anesthesia. Oral and mucosal herpes occurred in eight patients (29.6%) in the PBC group but not in the PRT group. Herpes resolved approximately 2 weeks after treatment with antiviral drugs. No severe complications, such as blindness, intracranial hemorrhage, or intracranial infection, occurred in either group. In addition, no deaths occurred during this study. A comparison of complication data is presented in Table 5.

**Table 5:** Comparison of complications between the two groups.

Complications	Group A	Group B	*p*-value
Facial hypoesthesia	100%	100%	
Mastication weakness	12.0%	44.4%	0.010
Headache	4.0%	33.3%	0.007
Nausea or vomiting	8.0%	22.2%	0.156
Dizziness	none	18.5%	0.024
Diplopia	none	18.5%	0.024
Facial herpes outbreak	none	29.6%	0.003

## Discussion

After 18 months of follow-up, the effective rates of pain control were 76.0 and 88.9% in the PRT and PBC groups, respectively. There were no significant differences in the postoperative efficacy between the two groups. The preoperative and postoperative VAS scores differed significantly between the two groups. All the patients developed hypoesthesia of the affected skin and mucosa immediately after surgery, which was a primary complication. Temporary postoperative discomforts, such as headache, dizziness, nausea, or vomiting, are associated with surrounding tissues or nerve irritation during the procedure. All the symptoms resolved after rest. Neither group had any intracranial hemorrhage, intracranial infection, or other serious complications, indicating that PRT and PBC are safe and effective.

TN is a painful disease that occurs worldwide. Currently, the pathological mechanism of its pain is unclear (11), and relapse has become a challenge in treating TN. Although MVD is still considered the most effective method, several patients opt for minimally invasive treatment. PRT and PBC have been repeatedly shown to be effective in treating TN. In our study, although a patient in the PBC group required a repeat of the initial procedure for recurrent TN, 52 patients with recurrent TN achieved satisfactory therapeutic effects over time. Previously, PBC was performed under general anesthesia; however, we believe local anesthesia is advantageous. The advantages of local anesthesia are simple operation, lower patient costs, and effective avoidance of severe complications such as trigeminal-cardiac reflex (TCR). TCR usually occurs when the trigeminal ganglion is punctured or pressed. The patient may experience a sudden drop in blood pressure and heart rate and may even be at risk of cardiac arrest. Although Belagava et al. noted that reducing blood pressure and heart rate in TCR was temporary, stopping ganglion stimulation resulted in a quick recovery (12). However, the risk remains high if the patient has a history of cardiovascular disease. When the ganglion is punctured, TCR may appear; however, it does not appear again after the completion of local anesthesia. Chowdhury et al. pointed out that the trigeminal ganglion blocks the reflex pathway after anesthesia; therefore, a vagus response cannot be formed (13). Therefore, we chose local anesthesia as the anesthesia mode.

One patient experienced decreased blood pressure, heart rate, sweating, and palpitations after anesthesia. Thus, the operation was terminated to ensure safety. This condition may be related to injecting a local anesthetic into the Meckel’s cavity when the puncture needle is too deep. After supportive treatment, the patient’s symptoms gradually disappeared without severe consequences. The injection of narcotic drugs into the blood or cerebrospinal fluid is a severe complication of local anesthesia, particularly when injected into the cerebrospinal fluid, which may inhibit the central nervous system. Therefore, precision is necessary in this regard.

Furthermore, CT-guided imaging has been widely used in PRT; however, it has not been widely used in PBC. Three-dimensional (3-D) imaging reconstruction produces more efficient and safer results than two-dimensional imaging (14). Preoperative three-dimensional imaging reconstruction of the skull base helps detect anatomic variations and avoid inadvertent injury to the peripheral neurovascular structures (15). Furthermore, the CT-guided approach reduces the number of operational adjustments and minimizes potential damage to the oculomotor and abducens nerves. Diplopia or keratitis is effectively eliminated, with historical incidences ranging from 1–4% and 5–12%, respectively (16, 17). The location of the foramen ovale and the depth of penetration of the cannula is visible, which gives the surgeon confidence and greatly improves patient safety. In addition, 3D reconstruction is more effective and accurate than other techniques in patients with perivale bone herniation and prevents successful intubation using conventional techniques. Our operations were all performed using CT, which differs from traditional surgery.

PRT and PBC showed satisfactory analgesic effects. However, patients experienced different outcomes postoperatively. Furthermore, headache, dizziness, nausea, and vomiting were the most common postoperative complications. Our results show that PRT resulted in a lower proportion of these symptoms. There were no facial herpes cases in the PRT group, and 8 cases were observed (29.6%) in the PBC group. Previous reports have described the occurrence of facial herpes following PBC. The rates reported by Berra and Tenser were 35.3 and 32.1%, respectively (18, 19). They suggested that the compression of the trigeminal ganglion activates the herpes virus present therein; however, the mechanism that causes the virus to be activated is unknown. To meet the surgical requirements, the concentration and dosage of anesthetic drugs differed for trigeminal ganglion block (PRT group 2%, 0.2 mL, PBC group, 1%, 0.5 mL). As there were more anesthetic drugs in the PBC group, the possibility of peripheral nerve block increased, and patients may have temporary postoperative diplopia. Although these symptoms are short-lived, they may cause more significant concern in postoperative patients.

Numbness is the most common complication of PRT and PBC. It has been reported that the incidences of postoperative numbness using these two methods are 98 and 90%, respectively (20, 21). All the patients in our study developed facial numbness after surgery (PRT group, 100%; PBC group, 100%). However, the numbness ranges differed owing to the different destruction modes. We can choose to destroy V1–V3 in PRT but not in PBC. Therefore, numbness may be more widespread in PBC. During PRT, we attempted to search for the V1 nerve by puncturing the foramen ovale, but none were observed. This may be related to the anatomical characteristics of the V1 nerve, and when the puncture is too deep, the tip of the needle may enter Meckel’s cavity and affect local anesthesia. Therefore, we added radiofrequency thermocoagulation to the supraorbital nerve in patients with TN of the V1 nerve. PBC can treat V1 pain by puncturing the foramen ovale without selecting the responsible nerve and may be recommended when multiple branches are involved, particularly in V1 neuralgia.

The key to PBC success is a pear-shaped image at the end of the catheter (22). The pear-shaped projection indicates that the balloon entered Meckel’s cavity. Nerve fibers that cause conduction pain can be effectively damaged. The oval shape indicates that the balloon did not enter the Meckel’s cavity, and the dumbbell type indicates that the balloon was too deep. Nevertheless, the latter two methods do not achieve satisfactory efficacy (23). In patients with recurrent pain, the anatomical structure may be damaged by previous treatment, which can cause difficulties in PRT and PBC. In the PBC group, one patient did not develop an ideal pear shape during the operation, and the pain was not controlled. After 10 days, PBC was performed. After repeated adjustments of the catheter position, a satisfactory figure was obtained, and the pain disappeared.

This was a prospective study with small sample size and a short follow-up period of 18 months. The long-term efficacy of PRT and PBC in treating recurrent TN requires a larger sample size, longer follow-up time, and a multicenter evaluation.

## Conclusion

CT-guided PRT and PBC under local anesthesia may be good options for treating recurrent TN. PBC may be recommended when multiple branches are involved, particularly in V1 neuralgia.

## Data availability statement

The original contributions presented in the study are included in the article/supplementary material, further inquiries can be directed to the corresponding author.

## Ethics statement

The studies involving humans were approved by the Ethics Committee of The Second Hospital of Hebei Medical University. The studies were conducted in accordance with the local legislation and institutional requirements. Written consent was obtained from all participants in this study.

## Author contributions

LX: Conceptualization, Data curation, Investigation, Methodology, Software, Writing – original draft, Writing – review & editing. XL: Conceptualization, Data curation, Formal analysis, Investigation, Writing – review & editing. HS: Conceptualization, Data curation, Investigation, Writing – review & editing. WH: Conceptualization, Data curation, Formal analysis, Methodology, Writing – original draft. LG: Conceptualization, Data curation, Methodology, Writing – review & editing. LW: Conceptualization, Data curation, Formal analysis, Methodology, Project administration, Writing – original draft. JL: Conceptualization, Data curation, Formal analysis, Investigation, Writing – review & editing. YR: Conceptualization, Data curation, Formal analysis, Investigation, Methodology, Project administration, Software, Writing – review & editing. YD: Conceptualization, Data curation, Investigation, Writing – original draft. GL: Conceptualization, Data curation, Methodology, Supervision, Formal analysis, Funding acquisition, Project administration, Writing – review & editing.
